# Supplementary figures and images for: The absence of core piRNA biogenesis factors does not impact efficient transposon silencing in Drosophila
Source: PLoS Biol. 2023 Jun 6;21(6):e3002099. doi: 10.1371/journal.pbio.3002099 (PMC10243637; doi:10.1371/journal.pbio.3002099)

# Supplemental Fig. S1

A

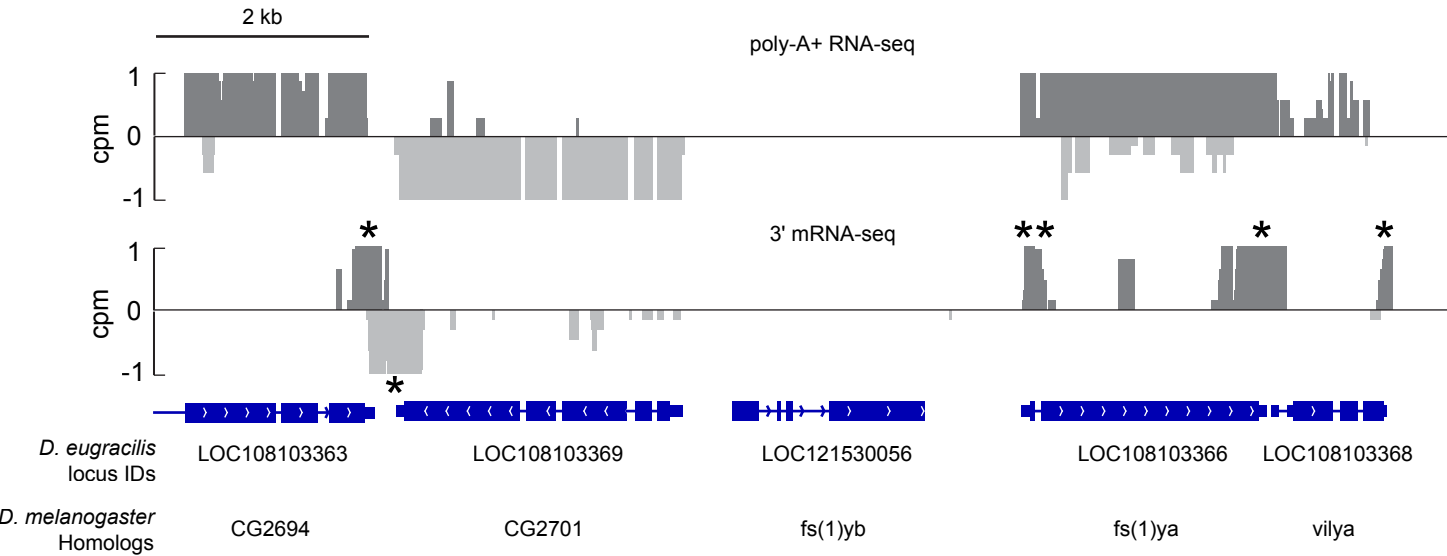

B

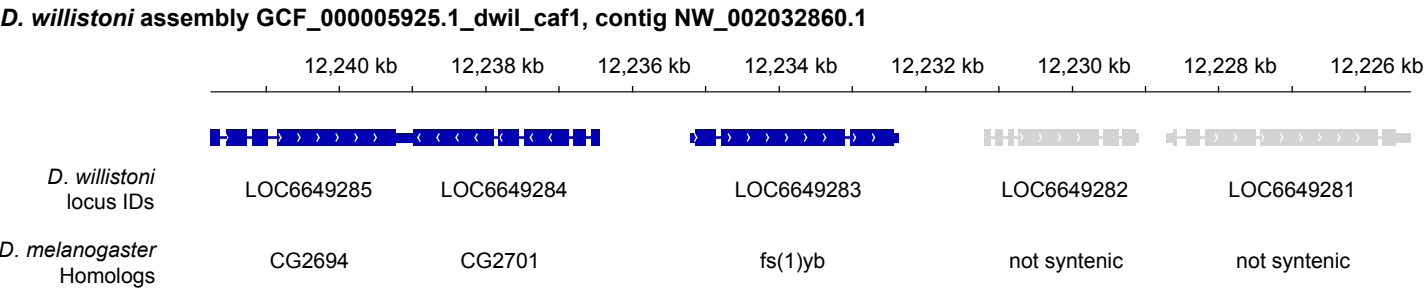

C

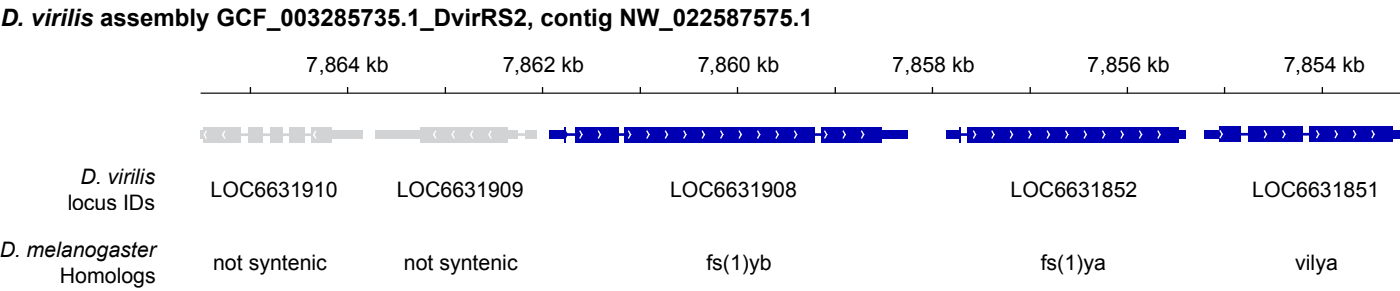

Supplement: S1 Fig — (A) Both poly-A+ RNA-seq and 3′ mRNA-seq show no expression of the pseudogene of yb in D. eugracilis. The coverage of neighbouring genes is shown in counts per million reads (CPM). Single and double asterisks indicate peaks of the 3′ mRNA-seq that correspond to canonical mRNA 3′ ends and a suspected internal priming, respectively. (B and C) Shown are the syntenic loci of yb gene in D. willistoni (B) and D. virilis (C) genomes. Genes that are found near the yb gene both in D. melanogaster and D. willistoni or D. virilis are coloured in blue, while genes that are found near the yb gene only in D. willistoni or D. virilis are coloured in gray. (PDF) [file pbio.3002099.s001.pdf]

# Supplemental Figure S2

A

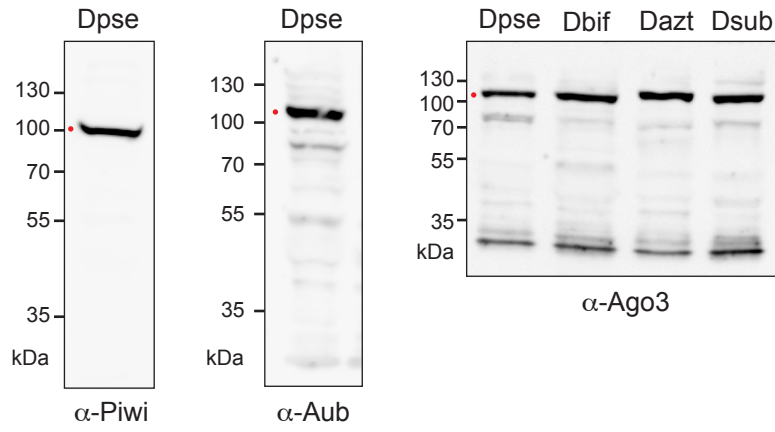

B

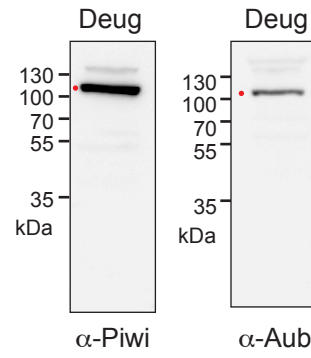

C

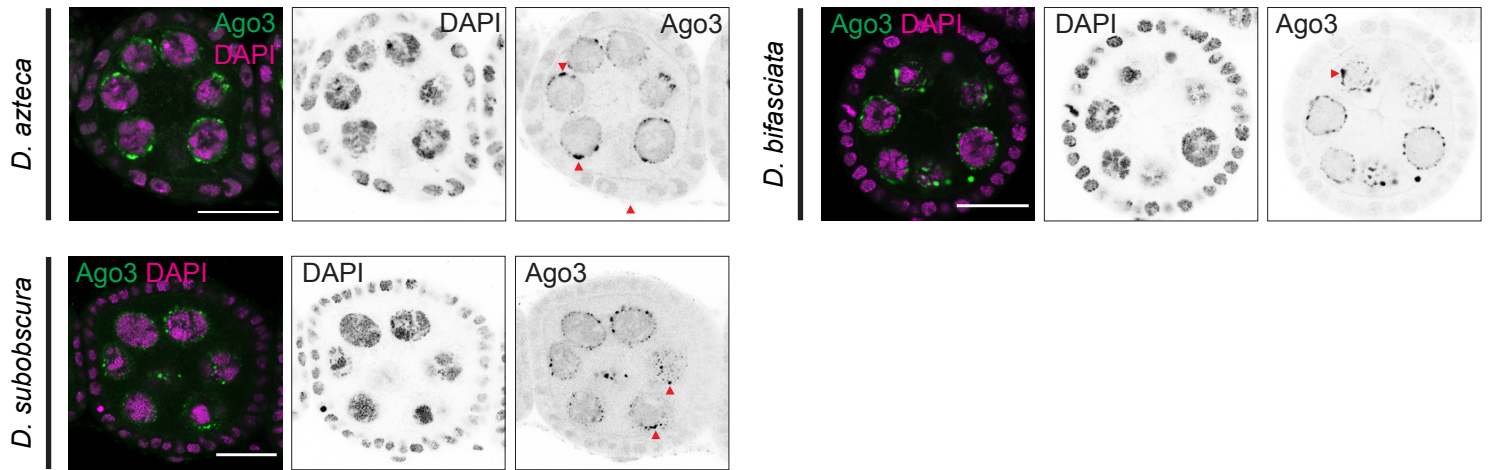

D

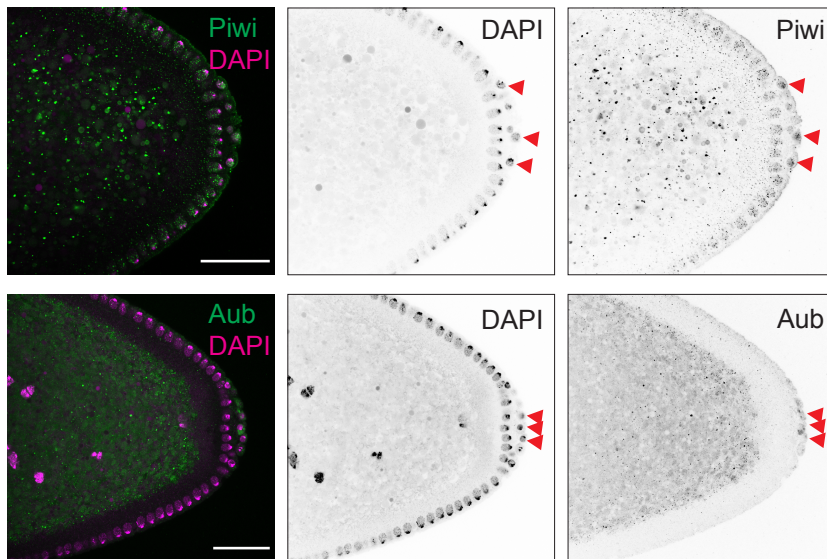

Supplement: S2 Fig — (A and B) Antibodies raised against peptides from D. pseudoobscura and D. eugracilis PIWI proteins were used in (A) and (B), respectively. The lysates were prepared from ovaries from indicated species and blotted for antibodies against Piwi, Aubergine (Aub), and Ago3. Dominant bands, as indicated by red dots, were detected at predicted sizes (100 to 110 kDa) in all cases. Species names are abbreviated as follows: Dazt, D. azteca; Dbif, D. bifasciata; Deug, D. eugracilis; Dpse, D. pseudoobscura; Dsub. D. subobscura. (C) Immunofluorescent stainings of (Ago3 in green and DAPI in magenta) of D. azteca, D. bifasciata, and D. subobscura egg chambers show a perinuclear localisation of Ago3 in all 3 species. Perinuclear granules, as indicated by arrowheads, are seen in all species. (D) Immunofluorescent staining of Piwi and Aubergine in D. eugracilis mid-blastoderm stage embryos showing the localisation of Piwi and Aubergine in the pole cells (marked by arrowheads) and in the zygotic somatic nuclei (Piwi only). Embryos of 1–1.5 h postfertilisation were stained. This is the stage after the nuclei reach the periphery of the blastoderm before the nuclear elongation and the onset of cellularisation. The bulk of zygotic transcription has not started in this stage. Scale bars = 20 μm. The underlying data can be found in S1 Raw Images file. (PDF) [file pbio.3002099.s002.pdf]

# Supplemental Figure S3

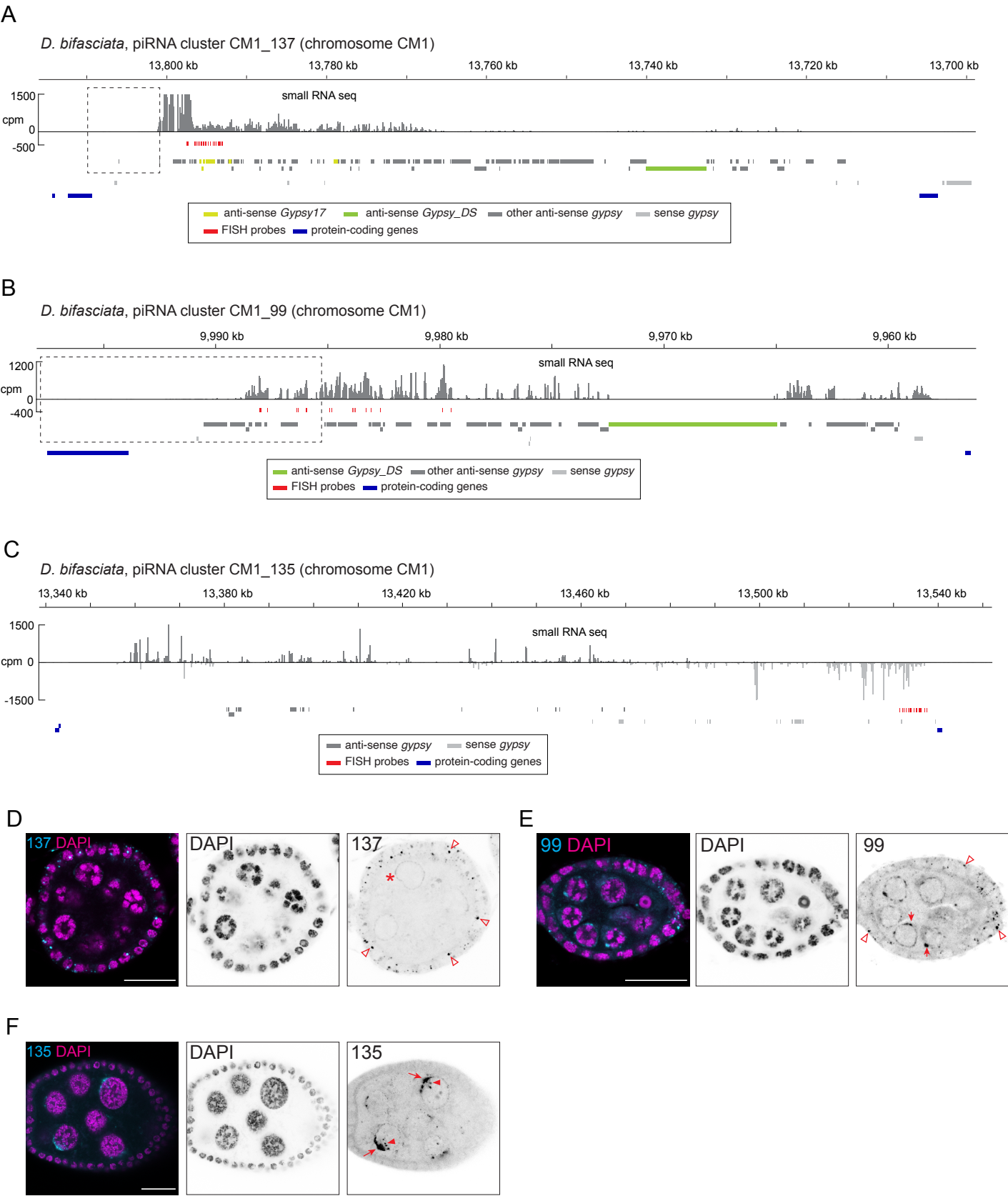

Supplement: S3 Fig — (A–C) Shown are the coverage of piRNA reads (>22 nt) in counts per million genome mappers (CPM) from the oxidised whole ovary small RNA library of D. bifasciata that uniquely mapped to the cluster regions. Sense and antisense reads are coloured in dark and light gray, respectively. Coloured bars indicate gypsy insertions predicted by RepeatMasker, annotated protein-coding mRNA exons, and the FISH probes. Dotted box in (A) and (B) indicate the putative transcription start sites of the cluster, for which magnified views are shown in S4B and S4C Fig. (D–F) RNA FISH against transcripts from the piRNA clusters. Somatic signals as indicated by open arrows are seen for the FISH against clusters CM1_137 (D) and CM1_99 (E), while the FISH against the cluster CM1_135 (F) only stains the germline cells. The FISH against CM1_137 weakly stained the germline at the nuclear periphery (asterisk), while distinct perinuclear puncta are seen in the FISH against CM1_99 and CM1_135 (arrows). Putative sites of transcription in the nuclei as indicated by arrowheads are only seen in CM1_135. (PDF) [file pbio.3002099.s003.pdf]

# Supplemental Fig. S4

A

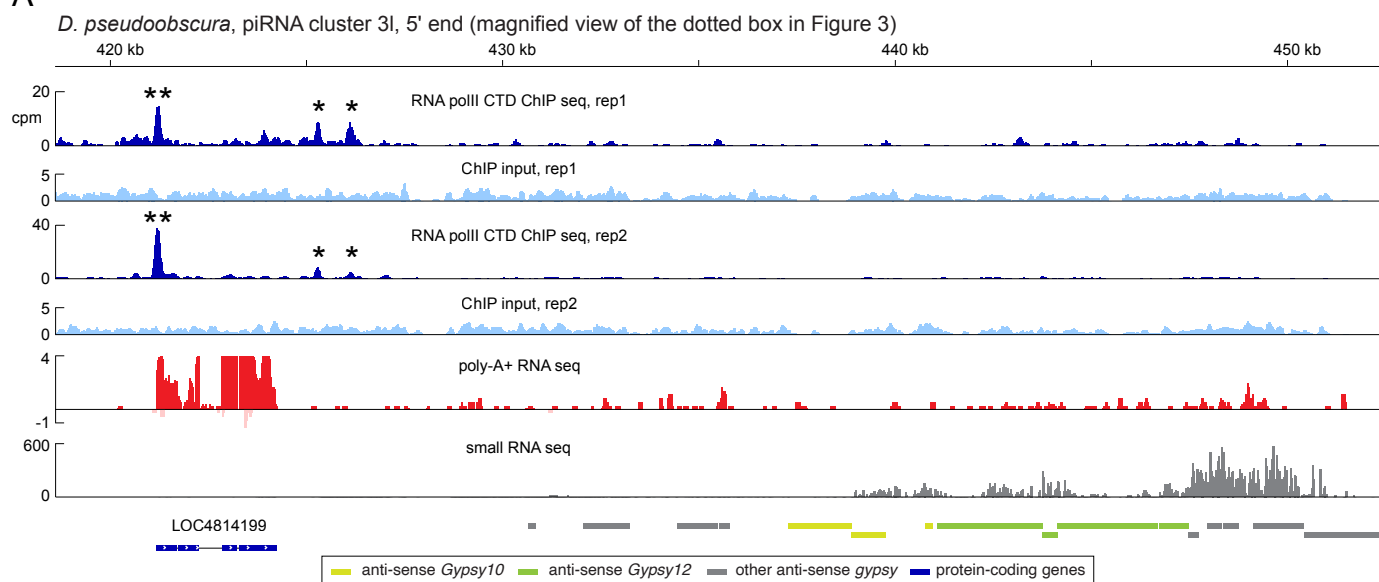

B

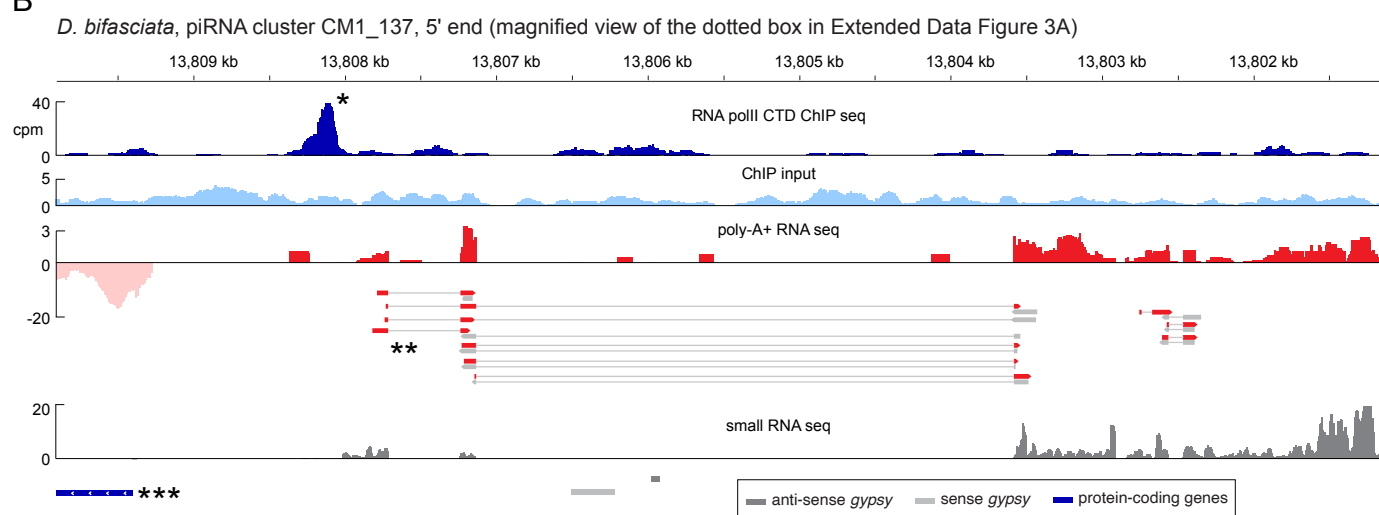

C

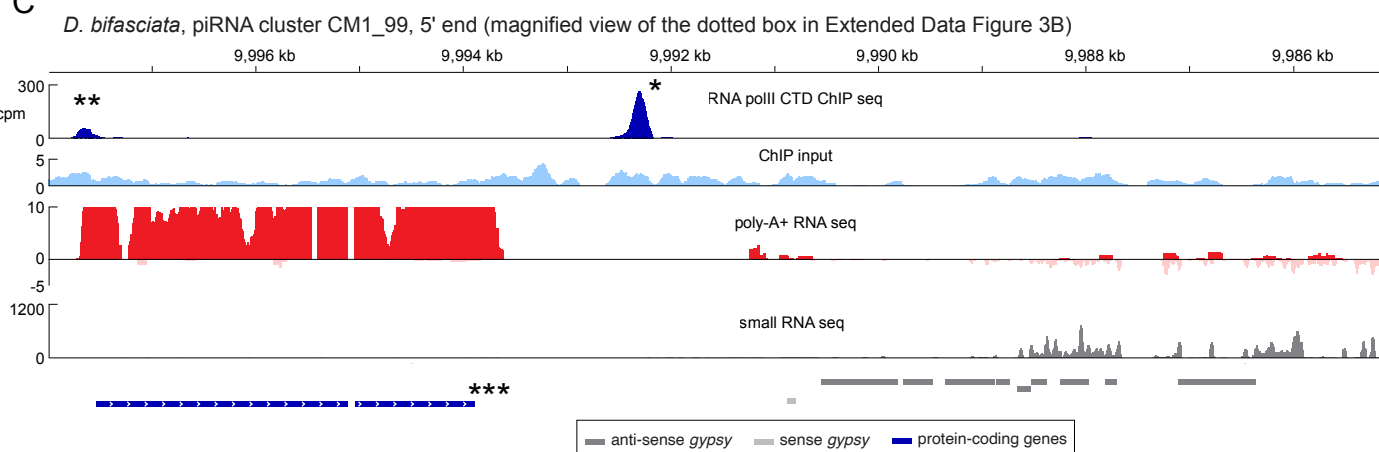

Supplement: S4 Fig — (A–C) Shown are the coverage of RNA polymerase II ChIP-seq (blue), and its input controls (light blue), poly-A+ RNA-seq (sense in red and antisense in pink), and the piRNA reads (>22 nt) at the 5′ end of the somatic piRNA clusters. Y-axes indicate counts per million genome mappers (CPM) for all tracks. RNA-seq read pairs that span introns are indicated as red and gray bars in pairs. Coloured bars at the bottom indicate gypsy insertions and annotated protein-coding mRNA exons. Peaks of RNA polymerase II in front of the clusters and at the neighbouring gene promoter regions are marked by single and double asterisks, respectively. Triple asterisks indicate protein-coding exons in D. bifasciata predicted by homology to D. pseudoobscura proteins. (PDF) [file pbio.3002099.s004.pdf]

# Supplemental Fig. S5

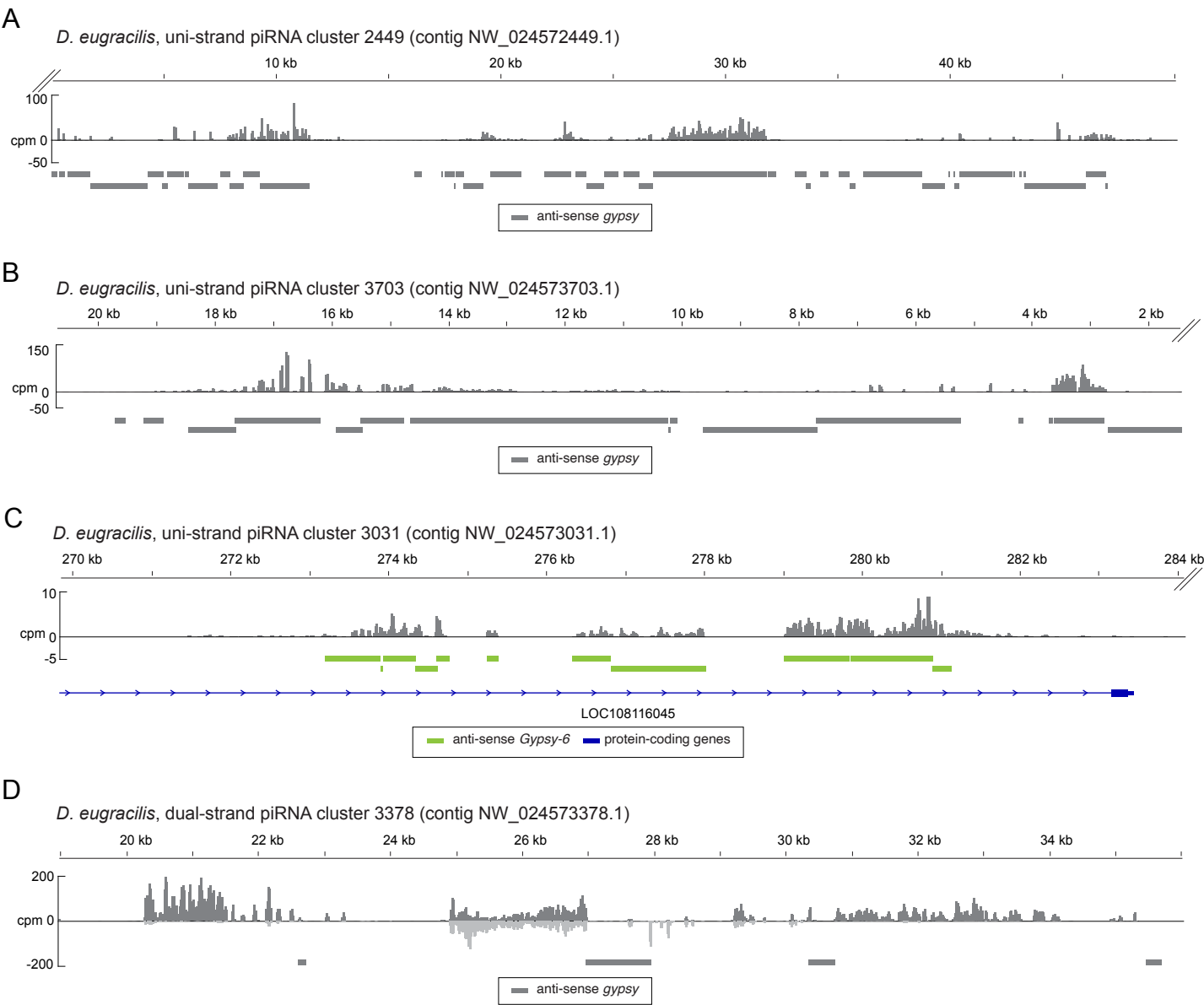

Supplement: S5 Fig — (A–D) Shown are the coverage of piRNA reads (>22 nt) in counts per million genome mappers (CPM) from the oxidised whole ovary small RNA library of D. eugracilis that uniquely mapped to the cluster regions. Sense and antisense reads are coloured in dark and light gray, respectively. Coloured bars indicate gypsy insertions predicted by RepeatMasker, and an annotated protein-coding mRNA exon. The entirety of the uni-stranded clusters could not be determined because they are found at the end (indicated by double dashed lines) of the chromosome contigs. (PDF) [file pbio.3002099.s005.pdf]

A

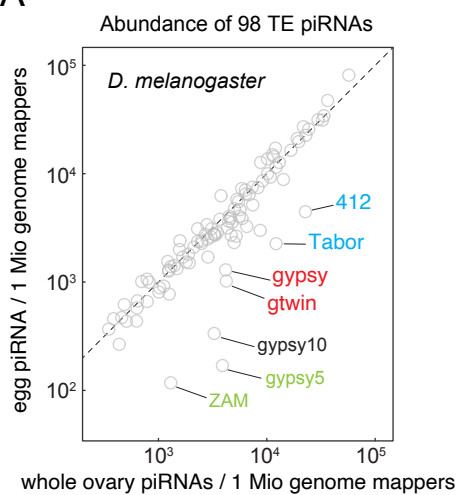

B

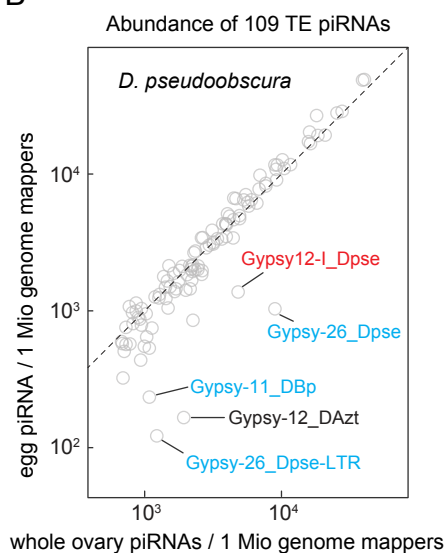

C

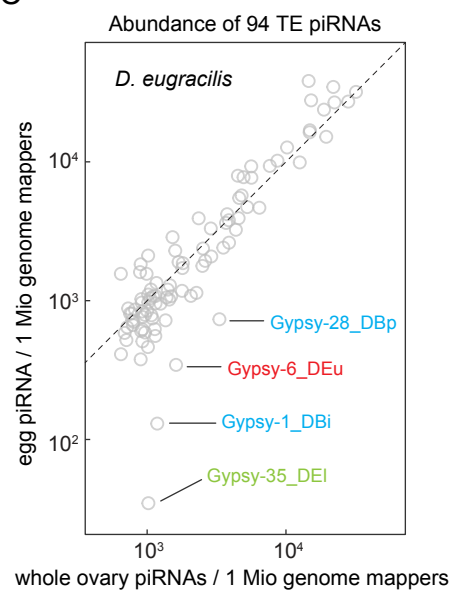

D

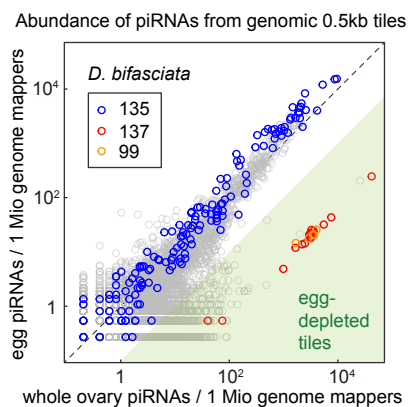

E

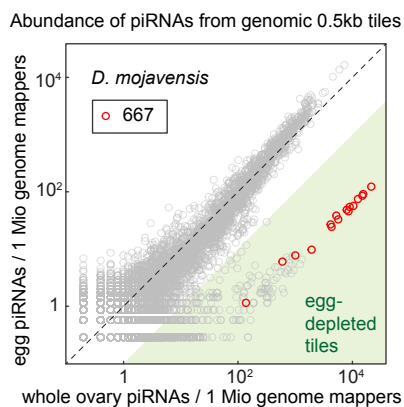

F

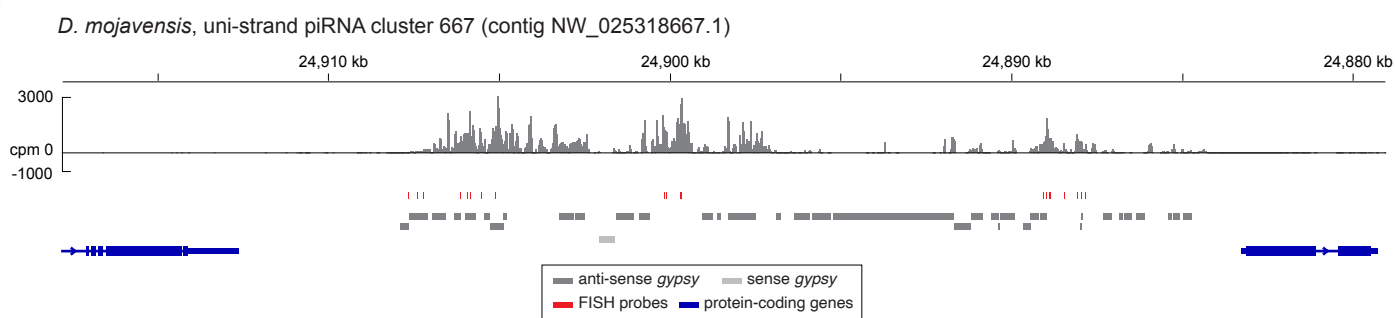

G

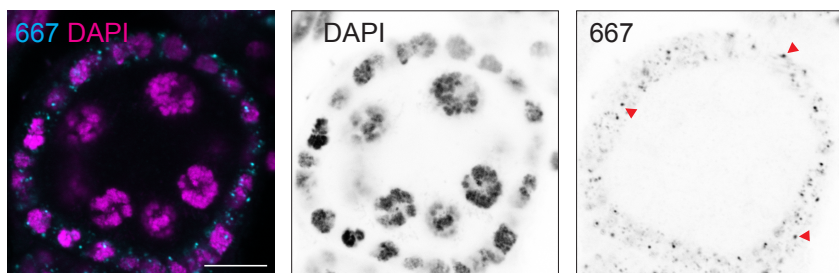

Supplement: S6 Fig — (A–C) Abundance of ovarian (X axes) and embryonic (Y axes) piRNAs mapping to individual transposons in D. melanogaster (A), D. pseudoobscura (B), and D. eugracilis (C) genomes. Transposons that expressed piRNAs more than 3 times in the ovaries than in the embryos are marked. Colours indicate families within the “errantiviridae/412” group of Ty3/Gypsy superfamily: red; group “Gypsy,” green; group “17.6,” cyan; group “412/mdg1,” and black; unclassified. (D and E) Scatter plots showing the abundance of piRNAs from the whole ovaries (X axis) and the eggs (Y axis) that uniquely mapped to the individual 0.5 kb tiles of the D. bifasciata genome in (D) and D. mojavensis genome in (E). The dual-stranded germline clusters are coloured in blue while uni-stranded somatic clusters are coloured in orange and red. Tiles that expressed piRNAs more than 10 times in the whole ovaries than in the embryos are shaded in green. (F) Shown is the coverage of piRNA reads (>22 nt) in counts per million genome mappers (CPM) from the oxidised whole ovary small RNA library of D. mojavensis that uniquely mapped to the cluster region. Sense and antisense reads are coloured in dark and light gray, respectively. Coloured bars indicate sense and antisense gypsy insertions predicted by RepeatMasker, annotated protein-coding mRNA exons, and FISH probes. (G) RNA FISH, showing the expression of the cluster 667 in the somatic cells of a D. mojavensis egg chamber. Focused signals in the somatic cells are indicated by arrowheads. Scale bars = 10 μm. (PDF) [file pbio.3002099.s006.pdf]

# Supplemental Fig. S7

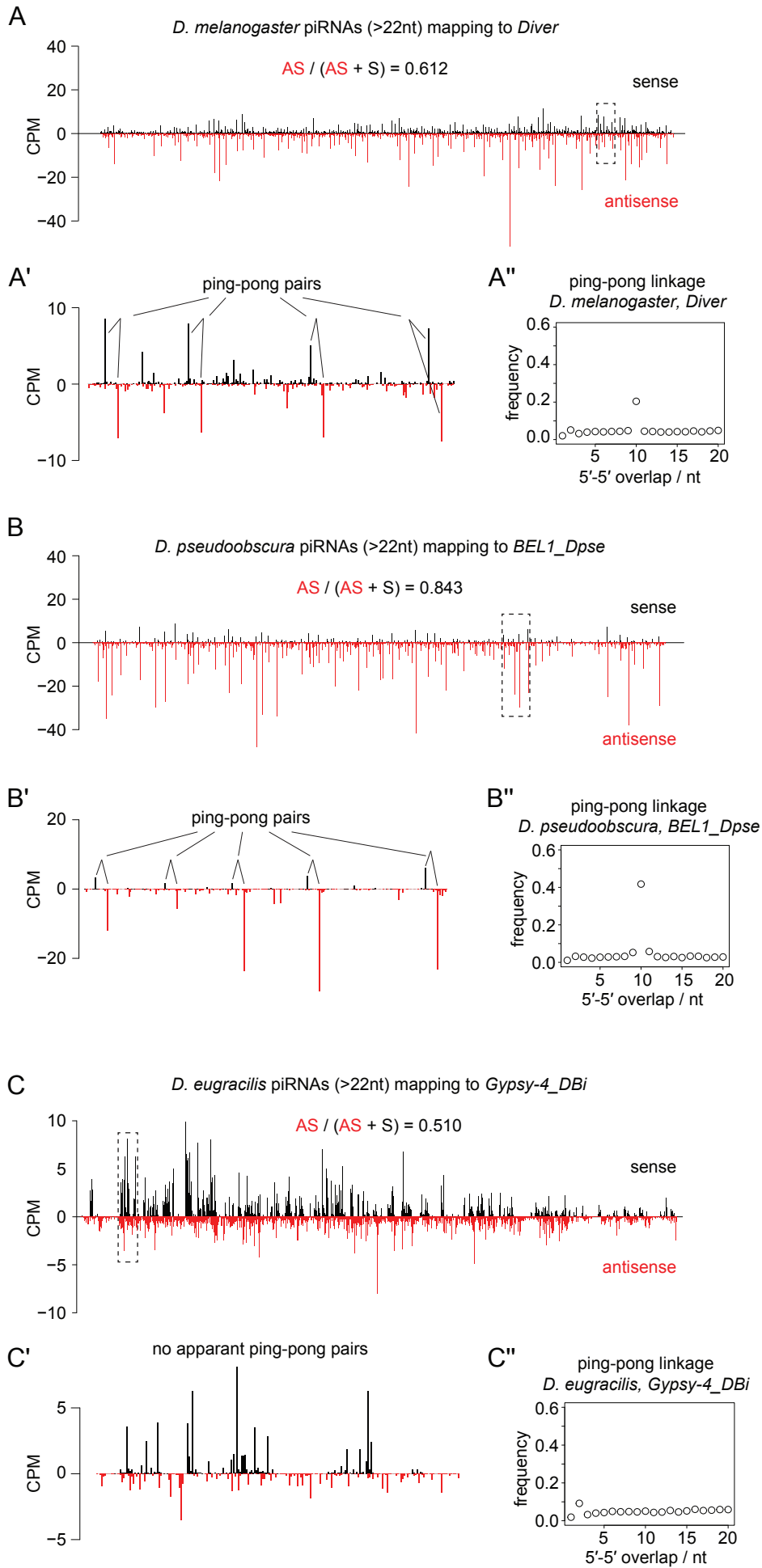

Supplement: S7 Fig — (A–C) Shown are the 5′ end coverage of piRNA reads (>22 nt) from D. melanogaster (top), D. pseudoobscura (middle), and D. eugracilis (bottom) ovaries mapping to indicated transposon sequences in counts per million genome mappers (CPM). Sense and antisense reads are coloured in black and red, respectively. Putative ping-pong pairs or absence of them are highlighted in the magnified view of the regions shown by dashed boxes (A’, B’, and C’). Frequencies of the 5′ overlapping bases between sense and antisense piRNAs are calculated where the characteristic 10 nt overlap is visible when there is a prominent ping-pong (A”, B”, and C”). The underlying data can be found in the S6 Data file. (PDF) [file pbio.3002099.s007.pdf]

# Supplemental Fig. S8

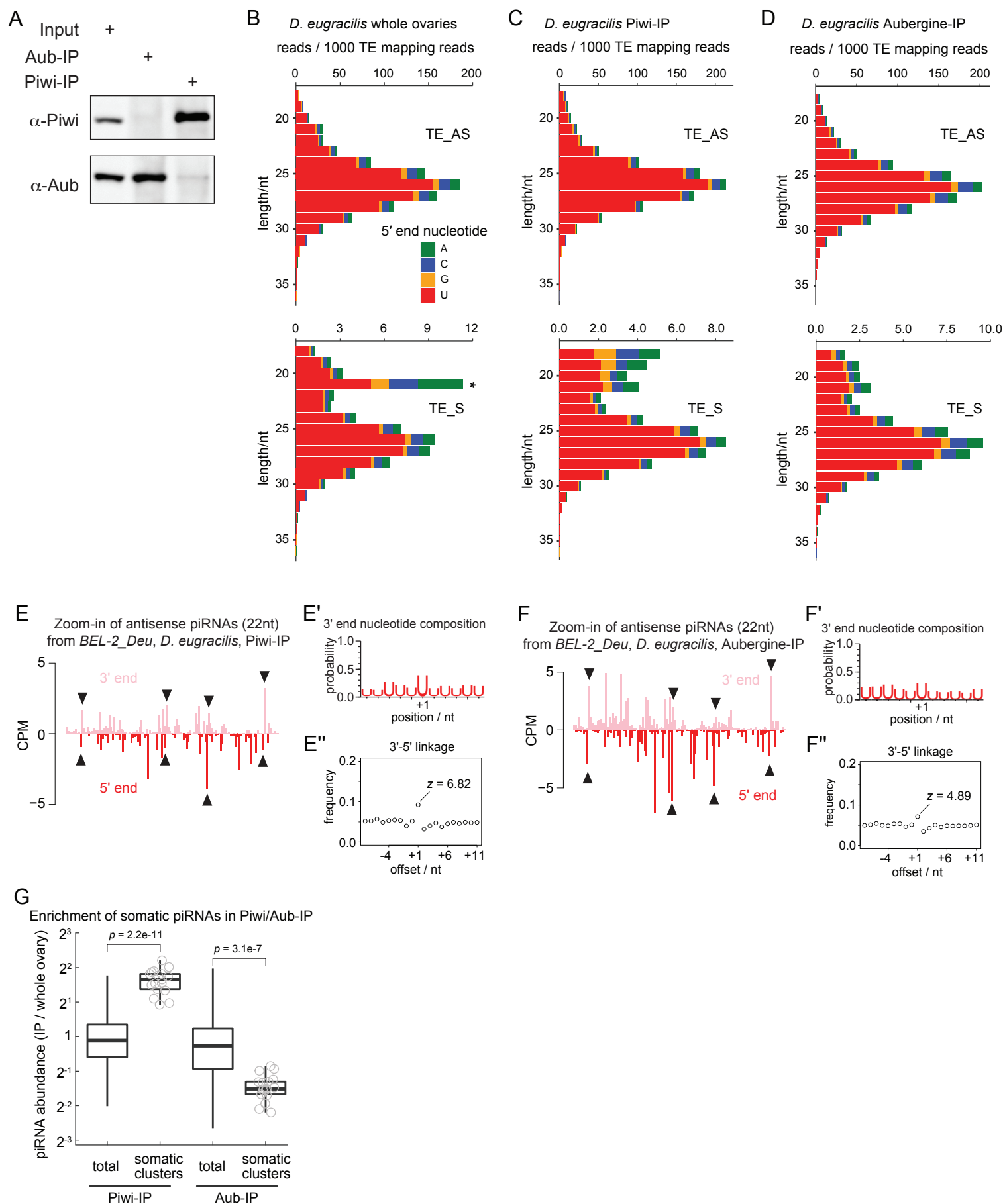

Supplement: S8 Fig — (A) A western blotting showing the specificities of antibodies against D. eugracilis Piwi and Aubergine (Aub). Input ovary lysates and the elutions after the immunoprecipitation (IP) are loaded and blotted against respective antibodies. (B–D) Size distribution of total ovarian (B), Piwi- (C), and Aubergine-bound (D) small RNAs mapping to transposon sense (bottom) and antisense (top) sequences. The proportion of nucleotides at the 5′ end is shown by different colours, showing the Uridine preference. The abundance is normalised to the total transposon mapping reads, showing that the majority of reads are antisense for all three libraries. There are very few putative siRNAs (21 nt, marked by asterisk) compared to piRNAs (24 to 29 nt), that are only detected in the sense reads and depleted in the IP libraries. (E and F) Shown are the 5′ and 3′ ends of Piwi- (E) and Aubergine-bound (F) piRNAs mapping to the antisense strand of BEL-2_Deu from the region indicated by a dashed box in Fig 4A. The 3′ and 5′ ends of piRNAs that are one nucleotide apart, hence the putative products of phasing, are marked by arrowheads. (E’ and F’) Shown are the frequencies of Uridines found at positions relative to the 3′ ends of piRNAs mapping to BEL-2_Deu. +1 corresponds to the immediate downstream nucleotide position. (E” and F”) Shown are the frequency plot of the 3′-5′ linkage of antisense BEL-2_Deu piRNAs. The z scores of the linkage position +1 are shown. (G) A box plot showing the relative abundance of genome-unique piRNAs from D. eugracilis mapping to 0.5 kb tiles, comparing the whole ovaries and Piwi- and Aubergine-bound pools. piRNAs mapping to the tiles from somatic clusters are enriched and depleted in Piwi- and Aubergine-bound pools, respectively. p-Values are calculated by Mann–Whitney U test. The underlying data can be found in S6 Data and S1 Raw Images files. (PDF) [file pbio.3002099.s008.pdf]

# Supplemental Fig. S9

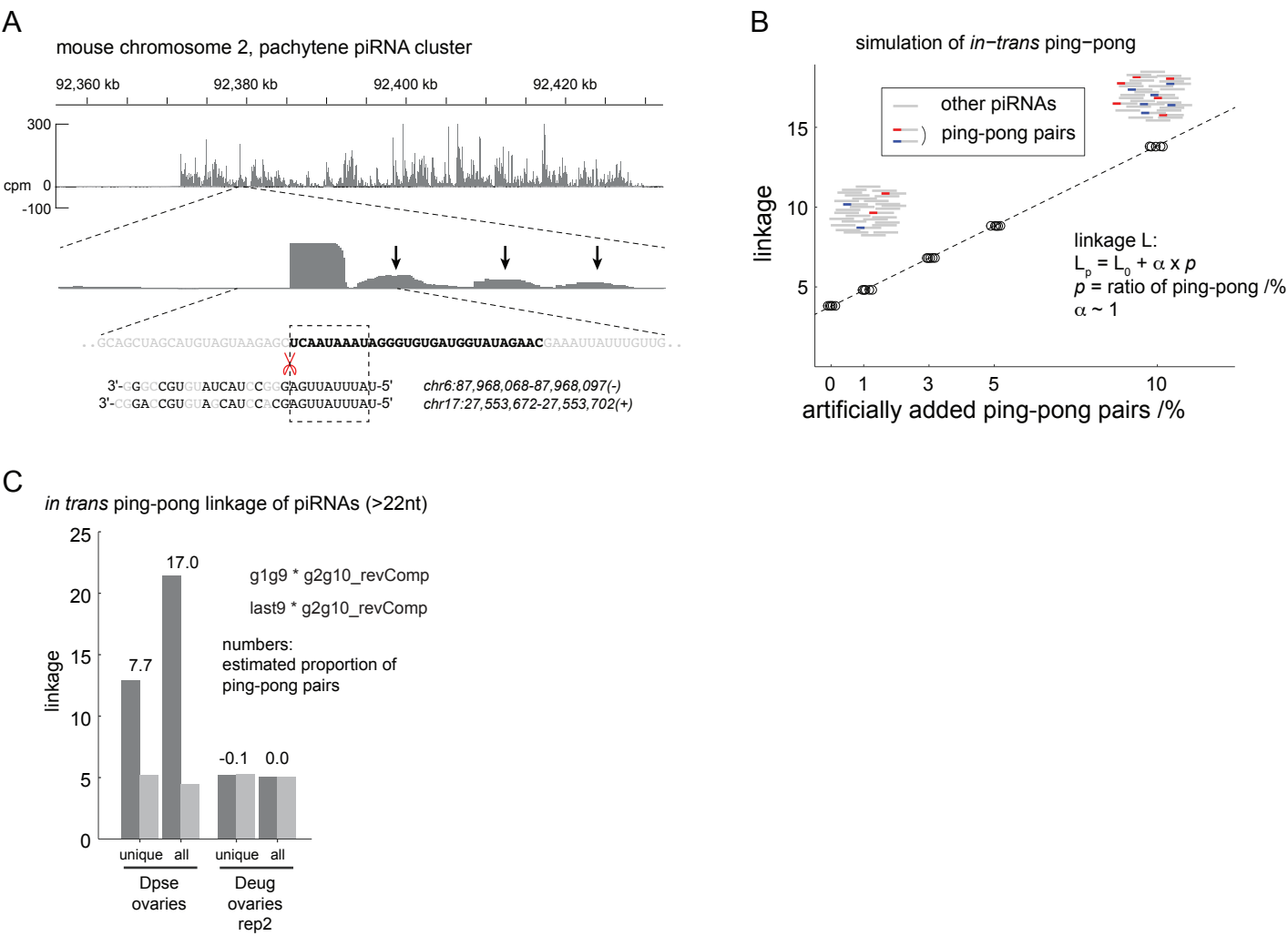

Supplement: S9 Fig — (A) Shown at the top is the coverage of piRNA reads (>22 nt) from a mouse pachytene piRNA cluster at chromosome 2 (PMID: 26115953). Shown at the bottom is an example of putative phasing events. The most abundant piRNA in this region is likely made as a result of slicing by piRNAs from distant genomic loci, which is followed by production of downstream piRNAs as indicated by arrows. (B) Shown are the in-trans ping-pong linkage values of simulated random piRNA pools with varying extent of ping-pong pairs artificially included (5 replicates each). The linkage value increases by one as the proportion of artificially added ping-pong pairs increases by 1%. (C) Shown are in-trans ping-pong linkage values of genome unique piRNAs and all piRNA mappers from D. pseudoobscura and the second replicate of D. eugracilis ovarian small RNA libraries. Estimated proportions of in-trans ping-pong pairs out of all piRNAs are shown in percentage. The underlying data can be found in the S6 Data file. (PDF) [file pbio.3002099.s009.pdf]
