# Supplementary material for: The absence of core piRNA biogenesis factors does not impact efficient transposon silencing in Drosophila
Source: PLoS Biol. 2023 Jun 6;21(6):e3002099. doi: 10.1371/journal.pbio.3002099 (PMC10243637; doi:10.1371/journal.pbio.3002099)
Supplement: S2 Data — (A) Amino acid sequences of Env proteins from Gypsy_DM, Gypsy12_Dpse, and Gypsy_DS are shown. Sequences of Gypsy12_Dpse and Gypsy_DS Env are taken from the insertions found in the D. pseudoobscura and the D.bifasciata genomes, respectively. (B and C) Alignments of Gypsy Env protein sequences made by Clustal Omega are shown. Functionally important motifs are shown in boxes; from the N-terminus, signal peptide, furin cleavage site, and the transmembrane domain [66,67]. Gypsy_DS Env protein was previously suspected to lack the peptide signal and the transmembrane domain [66]. However, the genomic insertion shown here retains those motifs. (DOCX) [file pbio.3002099.s011.docx]

Supplementary Data S2

A.

>GYPSY_I_3p

MMFIPLVVANARITDFSHANYIPVLDGDVLVFEQRDLLKHSSNLSEYASMIDETQKLSESFPHSHMRKLLEVDTDHLRTLLSVLKVHHRIARSLDFLGTALKVVAGTPDATDLFKIKITEAQLVESNSRQIAINSETQKQINKLTDTINKVINARKGDLVDTPHLYEALLARNRMLSTEIQNLILTITLVKSNIINPTILDHADLKPLVEQDTPIVSLIEASKIRVLQSENSIHILIAYPRVKFSCKKVAVYPVSHQHTILRLDEDTLAECEHDTFAVTGCTDTTHFTFCERSRRETCVRSLHAGNAAQCHTQPSHLREINPVDDGVVIINEAAAHVSTDGSPETLIEGTYLVTFERTATINGSEFVNLRKTLSKQPGIVRSPLLNIVGHDPVLSIPLLHRMSNENLHSIQNLMDDVESEGSPRLWFVAGVVLNFGLIGSLALYLALRRRRASREIQRTIDTFNMTEDGHKLEGG

GCF_009870125.1_UCI_Dpse_MV25 (NC_046679.1 30539870 30541304 -)

>Gypsy12_Dpse_env_30541304

MIILLVALVNARITDYSHSDYVPILDGDILVWDEINYLRHSTNLTDYERMADETANLTEMFPQSHMRKLLVVDTDHIRNMLATISVHHRVARSLNILGSVLKVVAGTPDADDLEKIRINEAQLIESNNRQISINSKSQEQINRLTDSVNKLLEAAKGKQIDSAHLYETLLARNRMLASELSNLMLTISLAKVNVINPVILDHDDLNSIFSNQLTNVIVTNILEVSKIKVFQSNSIIHFVIQFPKIKYICKKITIFPVAHNGTVLRLDDNIVADCNDQIVTVSDCKQTTTTTFCETSTKDSCAQGLYSGGVAHCQSQPSHLSAITLVDDGIIIINDHPAAVSFDGSAALNISGTHLITFNDYAVINGSRYQNRKNVQSRYPGVASSPLLNVTEHKRVLSLPFLHQLSEENLNFIKEIKEEVSSRSRPIFAFCLGLGICGLVCGMAMLRLYLTKKRDARQINGLMARLSAPGTATAQGGE

GCA_009664405.1_UCBerk_Dbif_1.0 (CM019041.1 9970475 9971896 +)

>Gypsy_DS_Dbif-env

LLVVLAAVNARITDFSHANYIPVLDGEVLVFDQRSYLRHSSNISEFISMIDETEKLSDSFPQSHMRKLLDVDTDHLRTLLSVLQVHHRFARSLDFLGTALKVVAGTPDASDFLKVRVTEAQLVESNSKQIIINSETQKQINRLTDTINKIISSRKGDLVDTPHLFETLLARNRILNTEIQNLILTITLAKANIVNPTILDHADLKSLIEQDTPIVSLLEASKIKVLQSENIIHILIAYPKVEFKCQKVSVYPVSHQQTILRLDEDTLAECERDTFAVTGCTVTTHNTFCERARRETCASSLHAGNTANCHTQPSHLNAIMPIDDGVVVINEATARVRTDDGAEVTVSGTFLITFERSAAINGTEFINLRKAPSKQPGTVRSPLLNIIGHDPALSIPLLHRMNINNLQSILDFKEEVIAAGSPKFWFAVGAVLNVGLICSFILFMALRRKRASLRIQKALDNFNMTEDGHHSEGG

B.

Gypsy_I_3p MMFIPLVVANARITDFSHANYIPVLDGDVLVFEQRDLLKHSSNLSEYASMIDETQKLSES 60

Gypsy12_Dpse_env_30541304 MIILLVALVNARITDYSHSDYVPILDGDILVWDEINYLRHSTNLTDYERMADETANLTEM 60

*::: :.:.******:**::*:*:****:**::: : *:**:**::* * *** :*:*

Gypsy_I_3p FPHSHMRKLLEVDTDHLRTLLSVLKVHHRIARSLDFLGTALKVVAGTPDATDLFKIKITE 120

Gypsy12_Dpse_env_30541304 FPQSHMRKLLVVDTDHIRNMLATISVHHRVARSLNILGSVLKVVAGTPDADDLEKIRINE 120

**:******* *****:*.:*:.:.****:****::**:.********** ** **:*.*

Gypsy_I_3p AQLVESNSRQIAINSETQKQINKLTDTINKVINARKGDLVDTPHLYEALLARNRMLSTEI 180

Gypsy12_Dpse_env_30541304 AQLIESNNRQISINSKSQEQINRLTDSVNKLLEAAKGKQIDSAHLYETLLARNRMLASEL 180

***:***.***:***::*:***:***::**:::* **. :*: ****:********::*:

Gypsy_I_3p QNLILTITLVKSNIINPTILDHADLKPLVEQDT---PIVSLIEASKIRVLQSENSIHILI 237

Gypsy12_Dpse_env_30541304 SNLMLTISLAKVNVINPVILDHDDLNSIFSNQLTNVIVTNILEVSKIKVFQSNSIIHFVI 240

.**:***:*.* *:***.**** **: :..:: :..::*.***:*:**:. **::*

Gypsy_I_3p AYPRVKFSCKKVAVYPVSHQHTILRLDEDTLAECEHDTFAVTGCTDTTHFTFCERSRRET 297

Gypsy12_Dpse_env_30541304 QFPKIKYICKKITIFPVAHNGTVLRLDDNIVADCNDQIVTVSDCKQTTTTTFCETSTKDS 300

:*::*: ***::::**:*: *:****:: :*:*:.: .:*:.*.:** **** * :::

Gypsy_I_3p CVRSLHAGNAAQCHTQPSHLREINPVDDGVVIINEAAAHVSTDGSPETLIEGTYLVTFER 357

Gypsy12_Dpse_env_30541304 CAQGLYSGGVAHCQSQPSHLSAITLVDDGIIIINDHPAAVSFDGSAALNISGTHLITFND 360

*.:.*::*..*:*::***** *. ****::***: * ** *** *.**:*:**:

Gypsy_I_3p TATINGSEFVNLRKTLSKQPGIVRSPLLNIVGHDPVLSIPLLHRMSNENLHSIQNLMDDV 417

Gypsy12_Dpse_env_30541304 YAVINGSRYQNRKNVQSRYPGVASSPLLNVTEHKRVLSLPFLHQLSEENLNFIKEIKEEV 420

*.****.: * ::. *: **:. *****:. *. ***:*:**::*:***: *::: ::*

Gypsy_I_3p ESEGSPRLWFVAGVVLNFGLIGSLALYLALRRRRASREIQRTIDTFNMTEDGHKLEGG- 475

Gypsy12_Dpse_env_30541304 SSRSRPIFAFCLGLGICGLVCGMAMLRLYLTKKRDARQINGLMARLSAPGTA-TAQGGE 478

.*.. * : * *: : : * * * * ::* :*:*: : :. . . :**

C.

GYPSY_I_3p MMFIPLVVANARITDFSHANYIPVLDGDVLVFEQRDLLKHSSNLSEYASMIDETQKLSES 60

Gypsy_DS_Dbif-env -LLVVLAAVNARITDFSHANYIPVLDGEVLVFDQRSYLRHSSNISEFISMIDETEKLSDS 59

::: *...******************:****:**. *:****:**: ******:***:*

GYPSY_I_3p FPHSHMRKLLEVDTDHLRTLLSVLKVHHRIARSLDFLGTALKVVAGTPDATDLFKIKITE 120

Gypsy_DS_Dbif-env FPQSHMRKLLDVDTDHLRTLLSVLQVHHRFARSLDFLGTALKVVAGTPDASDFLKVRVTE 119

**:*******:*************:****:********************:*::*:::**

GYPSY_I_3p AQLVESNSRQIAINSETQKQINKLTDTINKVINARKGDLVDTPHLYEALLARNRMLSTEI 180

Gypsy_DS_Dbif-env AQLVESNSKQIIINSETQKQINRLTDTINKIISSRKGDLVDTPHLFETLLARNRILNTEI 179

********:** **********:*******:*.:***********:*:******:*.***

GYPSY_I_3p QNLILTITLVKSNIINPTILDHADLKPLVEQDTPIVSLIEASKIRVLQSENSIHILIAYP 240

Gypsy_DS_Dbif-env QNLILTITLAKANIVNPTILDHADLKSLIEQDTPIVSLLEASKIKVLQSENIIHILIAYP 239

*********.*:**:*********** *:*********:*****:****** ********

GYPSY_I_3p RVKFSCKKVAVYPVSHQHTILRLDEDTLAECEHDTFAVTGCTDTTHFTFCERSRRETCVR 300

Gypsy_DS_Dbif-env KVEFKCQKVSVYPVSHQQTILRLDEDTLAECERDTFAVTGCTVTTHNTFCERARRETCAS 299

:*:*.*:**:*******:**************:********* *** *****:*****.

GYPSY_I_3p SLHAGNAAQCHTQPSHLREINPVDDGVVIINEAAAHVSTDGSPETLIEGTYLVTFERTAT 360

Gypsy_DS_Dbif-env SLHAGNTANCHTQPSHLNAIMPIDDGVVVINEATARVRTDDGAEVTVSGTFLITFERSAA 359

******:*:********. * *:*****:****:*:* **.. *. :.**:*:****:*:

GYPSY_I_3p INGSEFVNLRKTLSKQPGIVRSPLLNIVGHDPVLSIPLLHRMSNENLHSIQNLMDDVESE 420

Gypsy_DS_Dbif-env INGTEFINLRKAPSKQPGTVRSPLLNIIGHDPALSIPLLHRMNINNLQSILDFKEEVIAA 419

***:**:****: ***** ********:****.*********. :**:** :: ::* :

GYPSY_I_3p GSPRLWFVAGVVLNFGLIGSLALYLALRRRRASREIQRTIDTFNMTEDGHKLEGG 475

Gypsy_DS_Dbif-env GSPKFWFAVGAVLNVGLICSFILFMALRRKRASLRIQKALDNFNMTEDGHHSEGG 474

***::**..*.***.*** *: *::****:*** .**:::*.********: ***
