# Supplementary material for: The absence of core piRNA biogenesis factors does not impact efficient transposon silencing in Drosophila
Source: PLoS Biol. 2023 Jun 6;21(6):e3002099. doi: 10.1371/journal.pbio.3002099 (PMC10243637; doi:10.1371/journal.pbio.3002099)
Supplement: S1 Raw images — Original raw images for the western blots shown in S2 and S8 Figs are shown. Regions that are used in the figures are indicated by red dashed boxes. (PDF) [file pbio.3002099.s016.pdf]

S1 Raw images

Antibodies against *D. pseudoobscura* PIWI proteins (Ovarian lysates, S2 Fig)

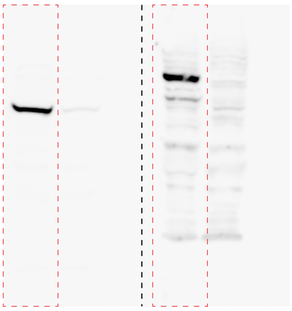

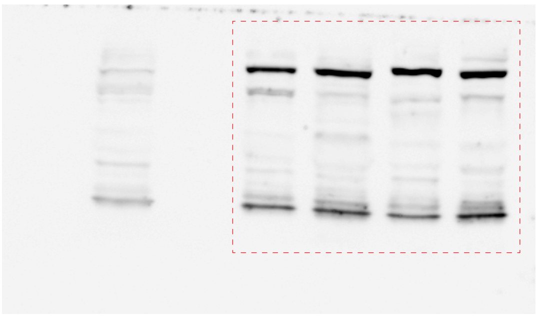

α-Piwi α-Aub α-Ago3

Antibodies against *D. eugracilis* PIWI proteins (Ovarian lysates, S2 Fig)

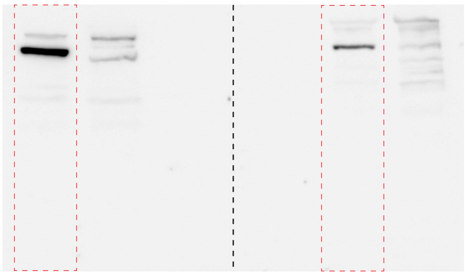

α-Piwi α-Aub

Antibody against *D. eugracilis* PIWI proteins (IP experiments, S8 Fig)

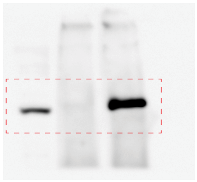

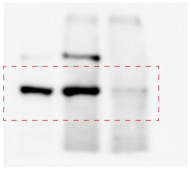

α-Piwi α-Aub
