# Supplementary material for: The absence of core piRNA biogenesis factors does not impact efficient transposon silencing in Drosophila
Source: PLoS Biol. 2023 Jun 6;21(6):e3002099. doi: 10.1371/journal.pbio.3002099 (PMC10243637; doi:10.1371/journal.pbio.3002099)
Supplement: Attachment — Submitted filename: Response-to-Reviewers.docx [file pbio.3002099.s017.docx]

Dear Editor and Reviewers,

Thank you for providing us with valuable comments to improve our manuscript. Below we provide point-by-point responses and the parts in the revised manuscript where you can find modifications. We believe that the revised manuscript has satisfactorily addressed all the comments.

Yours sincerely,

Rippei

A request to the Editor:

We would like to include my additional affiliation “The Shine-Dalgarno Centre for RNA Innovation”.

Responses to Editorial requests:

(A) Your manuscript is being considered as a Discovery Report, which has a maximum of 4 main figures. At this stage, we ask that you please reduce the number of main figures to 4, either by combining figure panels in the main figures or by moving some of the figures to the Supplementary.

**Response:** We now merged the previous Figures 1 and 2, and Figures 3 and 4 into single figures. Texts and Figure legends have been modified accordingly.

(B) We would like to suggest the following modification to the title, to make it more compelling for our broad readership:

“Proteins thought to be essential for piRNA biogenesis are absent in several Drosophila species that nevertheless display effective transposon restriction”

**Response:** We felt that the suggested title might be too long. We are inclined to keep the original title that succinctly delivers the message. Alternatively, we propose the following tile: “The absence of core piRNA biogenesis factors does not impact efficient transposon silencing in Drosophila”

(C) You may be aware of the PLOS Data Policy, which requires that all data be made available without restriction…

(E) We require the original, uncropped and minimally adjusted images supporting all blot and gel results reported in the following figures…

**Response:** We provided a table that contains all the raw numerical data (raw_numerical_data.xlsx). Also note that the previous supplemental Figure 10 contained raw western blotting images. We renamed this file as “raw-western-blotting-images.pdf”. Supplemental files are also renamed as suggested.

(D) Please also ensure that each of the relevant figure legends in your manuscript include information on *WHERE THE UNDERLYING DATA CAN BE FOUND*, and ensure your supplemental data file/s has a legend.

**Response:** All figure and table legends are found in the main manuscript file.

Responses to Reviewers’ comments:

Reviewer #1
**1.** I believe that the evolutionary angle of this story is very much understated. For somebody who is not well familiar with the piRNA field, it might seem at a first glance like an anecdotal observation. Species lose genes all the time so what is so striking about losing Yb or Ago3? In that sense it is important to emphasize the fact that flies with no intact piRNA system tend to be sterile. Furthermore, putting these findings in the context of evolutionary "arms-race" between hosts and transposons is also important. Transposons evolve very fast, just like viruses, and hence the evolution of the systems that defend against them also tend to be very fast. In this context, losses are more frequent. Relevant papers to cite in this context include (but there are others as well):
Luo et al. 2020 BMC Evol. Biol. 20: 14; Aravin et al. 2007 Science 318: 761-764 (already mentioned by the authors but in a different context); Cosby et al. 2019 Genes Dev. 33: 1098-1116. The paper will benefit from addressing this topic in more detail in the discussion as it will make the importance of the results clearer to a broader readership.

**Response:** We agree with the suggestion from reviewer #1. We have added a section in the discussion (“Evolutionary drivers of the loss of essential piRNA biogenesis factors”) to discuss about potential mechanisms that led to the loss of *yb* and *ago3* in light of the evolutionary arms-race between the TEs and the piRNA pathway.

**2.** The authors mention in the introduction very briefly that "Both ping-pong and phasing mechanisms are evolutionarily highly conserved, present in sponge and hydra species all the way to humans". First of all, the cited papers do not deal with "hydra species", but with cnidarians and actually Grismson et al. who the authors cite (Ref. 16) show data for Nematostella vectensis, which is a sea anemone (A very far relative of hydras. Same phylogenetic distance as between human and Drosophila). This is an important point as it helps putting the results in an appropriate evolutionary context: the ping pong cycle probably appeared in the last common ancestor of all animals. Thus, it deserves a more serious mention and might fit better in the discussion.  Relevant citations that should be included in addition to Grimson et al 2008 are: Juliano et al. 2014 PNAS 111: 337-342; Praher et al. 2017 RNA Biol. 14: 1727-1741 and Calcino et al. 2018 BMC Evol. Biol. 18: 160.

**Response:** Thank you for pointing this out. We have now corrected the text in the third paragraph of introduction and have also cited Lim et al. 2014 Dev Biol, which has described the ping-pong pathway in hydra species. We have furthermore added emphasis to the conservation of ping pong in the discussion, under the section “Phased piRNAs can be made without ping-pong/slicer activity (please also note that we changed the caption title to better describe the content.)”.

3. Continuing the previous point, an important paper to mention and briefly discuss in the context of the current results is that of Lewis et al. (2018 Nat. Ecol. Evol. 2: 174-181). This paper describes the finding of somatic piRNAs in arachnids (a taxon of non-insect arthropods) and the presence and absence of the ping-pong cycle in some of the tested species. This can provide an example for another adaptation of the piRNA system over a different phylogenetic scale I the same group (arthropods).

**Response:** We agree with the reviewer that the findings of the Lewis et al. Paper is an important point to discuss in the context of our findings relating to ping-pong biogenesis and have discussed it under the section “Phased piRNAs can be made without ping-pong/slicer activity”.

4. I know that some researchers working on flies feel like Drosophila is an English word, but in fact it is in Latin. Hence, it should be italicized like any other scientific Genus name. This should be done throughout the text.

**Response:** While we appreciate different styles of nomenclature surrounding the italicization of genus and species, we follow a broadly used notation system in Australia (as well as broadly in the United States) where the genus is only italicized if followed by the species.

5. I feel that the last sentence of the abstract is a bit missing the truly striking result of this study: the loss of the ping-pong cycle (and Ago3) in Drosophila eugracilis and puts the focus on the loss of Yb in multiple species. Please consider to rephrase it so it will account for these findings better.

**Response:** We agree with the reviewer and now changed the last sentence of the abstract.

6. The authors write "We could not determine the cell-type specific expression of D. eugracilis clusters by FISH as they expressed much fewer piRNAs per kilo bases than clusters from the other species (data not shown)." I doubt that "data not shown" is a valid statement in 2023 in a journal that promotes full transparency. Please consider showing the data as supplementary.

**Response:** We agree with the reviewer on the data transparency. However, in this case, we do not see the value of showing a negative result, as we don’t have an appropriate positive control to compare against. However, we noticed that “data not shown” is wrongly cited in this case. Supplemental Fig. S5 shows that the somatic piRNAs are less abundant per kilo base in *D. eugracilis* compared to *D. melanogaster* and *D. pseudoobscura* (Fig. 2 and Supplemental Fig. S3). We have modified the text accordingly.

7. Supplementary Figure 10 is in my opinion quite important in order to understand better the findings of this paper. Thus, I do not understand why it is a supplementary Figure. Please consider to make it into a main figure or combine it with an existing main figure.

**Response:** We agree with the reviewer. We have now inserted it in Figure 4.

Reviewer #2
1. To complete the analysis of conserved genes in different Drosophila species, the authors should include conservation analysis for zucchini, the nuclease that generates primary and phased piRNAs, and vasa, the RNA helicase that coordinates ping-pong.

**Response:** We agree that the conservations of other genes need to be shown. We have therefore included a homology search for the PIWI proteins, Vasa as well as Zucchini in the Supplemental Table S1. The method section was also modified to include these searches.

2. Introduction: "there are two distinct modes of piRNA biogenesis…": to provide a complete picture for a general audience, the authors should discriminate between primary piRNA biogenesis and phasing, which is a sub-pattern of primary biogenesis or ping-pong induced ('tertiary piRNAs'). In addition to references 12 and 13, Nishimasu et al, and Ipsaro et al Nature 2012 should be cited. This would be also helpful throughout the article, as non-phased primary piRNAs in addition to phased pairs are clearly visible in Fig. 6E

**Response:** We appreciate the suggestions and now included the two citations in the introduction. We defined the “phasing” in the manuscript to be the mechanism that depends on Zucchini/MitoPLD. The term “primary biogenesis” has been traditionally used to describe non-ping-pong piRNA biogenesis. To our knowledge, non-ping-pong piRNA production is all dependent on Zucchini in Drosophila. Therefore, “phasing” and “primary biogenesis’ are interchangeable when the Drosophila piRNA pathway is in question. We inserted a note in the introduction that “phasing biogenesis” is also called “primary biogenesis”.

Much of phased/primary piRNAs are produced after the slicing in Drosophila germline. Therefore, we prefer to use the term “phasing” to avoid confusion.

The apparent lack of head-to-tail arrangement seen, for example, in Fig. 4E is likely due to overlapping phases of piRNA production and the potential resection of piRNA 3’ ends after Zucchini cleavage (Mohn, Science, 2015; Han, Science, 2015). Genetic evidence shows that the depletion of Zucchini abolishes somatic piRNAs and only leaves ping-pong piRNAs in the germline of *D. melanogaster* (for example, Handler, EMBO J, 2011).

3. Fig. 1B: the authors mention a >10-fold decrease in flamenco-derived piRNAs and a > 100-fold increase in gypsy transposons in the results.  The corresponding figure only shows the gypsy TE data and forgot to include the flam data.  The authors should add the flam data.

**Response:** Thank you for pointing this out. Ishizu et al. 2019 observed a marked reduction of the *flam* piRNAs in Yb-depleted OSCs. However, they did not state the fold change. Therefore, we removed “>10-fold” from this part. Please also note that we provided an estimated fold-change later in the results section (Figure 3E).

4. Fig. 4A-D: The authors focus on similarities between Armi staining in different Drosophilids in the text. However, the figures show significant differences too. For example: the ratio of germ cell compared to follicle cell staining for Armi in D. Azteca and D eugracilis.  These might be differences in antibody affinities or might have biological significance.  The authors should comment on this.

**Response:** Thank you for pointing this out. Armitage gene has been duplicated in the *obscura* group genomes. It is possible that different Armitage proteins with different sensitivities to the antibodies are expressed in the germline and the somatic cells of *D. azteca*. The stronger germline staining of Armitage in *D. eugracilis* likely reflects on the dominance of phasing in the germline. We included these statements (only referring to the later section for *D. eugracilis*) in the result section “**Somatic biogenesis bodies are present in the *obscura* group species and *D. eugracilis***.”

5. Chapter "Specialized piRNA biogenesis for gypsy is evolutionarily conserved in Drosophila".  In addition to the co-factors for primary piRNA biogenesis in the gonadal soma (Armitage and Yb), the authors should include conservation analysis for the processing nuclease itself (Zucchini).

**Response:** Addressed in comment #1

6. Chapter: "Absence of ping-pong piRNAs in D. eugracilis".  In support of their data revealing tolerance for loss of ping-pong in some D. eugracilis, the authors should discuss Vrettos et al., LSA 2020, who suggested that ping-pong amplification is required for germplasm and the formation of a new germline but largely dispensable for fertility in D. melanogaster.

**Response:** Vrettos et al showed that the ping-pong is intact in the Csul mutant where Aubergine-dependent germ plasm formation is specifically affected. This observation suggests that ping-pong-independent function of Aubergine is required for the germ plasm formation. We now briefly added this ping-pong-independent role of Aubergine in the discussion section “**Phased piRNAs can be made without ping-pong/slicer activity.**”

We kept this part short in the discussion because we intend to emphasize the biological functions of ping-pong piRNAs known in *D. melanogaster* to contrast the lack of ping-pong in *D. eugracilis*. Other roles of Aubergine that are independent of its function in ping-pong may of course be conserved in *D. eugracilis*.

7. Chapter: "Piwi and Aub receive phasing piRNAs in D. eugracilis". This should read "…primary and phased piRNAs".  The authors should extend their phasing analysis to provide the following numbers: What fraction of piRNAs showed a signature of 'phased piRNA pairs' (Coincidence of a piRNA 3' end with a piRNA 5' end)? What fraction shows three or more phased piRNAs (Coincidence of a piRNA 3' end with the 5' end of a piRNA that has a 3' end that coincides with yet another 5' end). In previous reports these numbers have been rather small with fractions <1 % for ping-pong-induced phasing.  The authors provide such numbers for mouse piRNAs later in the manuscript but numbers for Drosophila data are missing.  Clearly defining what is meant by 'phasing' (pairs versus longer trails) and clarifying the prevalence of phased signatures in primary piRNAs and ping-pong-induced 'tertiary' piRNAs is essential to evaluate the importance of these signatures for piRNA biogenesis.

**Response:** As we mentioned in response to the comment #2, “phasing biogenesis” is interchangeably used in place of “primary biogenesis” to describe Zucchini-dependent piRNA biogenesis in Drosophila.

We calculated the linkage of the nucleotide positions of piRNA 3’ and piRNA 5’ for a selected TE (Fig. 4E) as a measurement of phasing propensity. The linkage is generally less remarkable compared to the ping-pong signature likely due to overlapping “phases” and the resection of piRNA 3’ ends (Mohn, Science, 2015; Han, Science, 2015). We cannot measure the true abundance of head-to-tail phasing unless we genetically perturb the mechanism of piRNA 3’ end resection, which is out of the scope of the current manuscript.

For a similar reason, the abundance of head-to-tail phasing of more than two piRNAs is difficult to measure. In the previous study where they measured such events (Mohn, Science, 2015), authors focused on the phasing events where the phasing is triggered and the site of initiation is clearly visible in protein-coding mRNAs. Due to the lack of systemic slicing-dependent phasing, it is practically very difficult to determine the nucleotide positions at which phasing starts.

We define “phasing” as Zucchini-dependent piRNA biogenesis throughout the manuscript. We introduced “phasing” as Zucchini-dependent and head-to-tail production of piRNAs in the introduction section. In the results section “**Piwi and Aubergine receive phasing piRNAs in *D. eugracilis*.**”, we explained that the propensity of phasing biogenesis can be measured by the linkage analysis of piRNA 3’ and 5’ ends and the Uridine bias of the immediate downstream nucleotide of the piRNA 3’ ends. We believe these are clear enough descriptions and well-accepted definition of the phasing mechanism.
We showed the genome-wide “in-trans” ping-pong linkage value for Drosophila ovarian piRNAs (Fig. 4). “in-trans” ping-pong linkage measures the sum of the canonical ping-pong and the slicing-mediated triggering events that lead to the phasing biogenesis. Relative contributions between the two can be inferred by comparing the values of genome-unique-mapping (mainly measuring the slicing-mediated triggering events) and genome-all-mapping piRNAs (including both the triggering and ping-pong events). We described these points at length in the results section.

8. "Phasing occurs without slicing by PIWI proteins in the D. eugracilis germline".  The authors should add that ping-pong independent phasing has also been observed for somatic piRNAs in D. melanogaster though at low level (Mohn et al., Science 2015; Han et al., Science 2015).  Statements for mouse pachytene piRNAs should be revised in the light of new data indicating the absence of efficient slicer-induced phasing and revealing an interesting tolerance for slicer activity without complementarity base-pairing across target nt 10-11 (Dowling et al., RNA 2023).

9. in the discussion the authors state: "phasing biogenesis requires a 'triggering' event…" -but does it? Efficient processing by the endonuclease zucchini has been shown to produce variable and densely packed piRNAs across precursors. Individual cleavage events could produce 3' and 5' ends for neighbouring piRNAs either simultaneously or from different precursor molecules.  Either event would appear as 'phased' signature in piRNA sequencing data.  The authors should explain the definition of 'phasing' and an observable signature in sequencing data and explain different possible mechanistic explanations.  They could discuss all these possibilities in support of their observation of variable 'phasing' signatures.

**Response to #8 and #9:** Yes, previous works showed that the lack of slicing does not completely abolish the phasing piRNA production in *D. melanogaster* germline. We stated that “nearly all germline phase piRNAs” are gone when both Aubergine and Ago3 are depleted. We believe this is sufficient to claim that an alternative mechanism should exist in the germline of *D. eugracilis* to support abundant phasing events. However, we agree that this is the case only for the germline and that phasing occurs independently of slicing in somatic cells of *D. melanogaster*. We corrected inaccurate statements in anywhere possible, for example, in the third last paragraph of the discussion.

As stated above, we define “phasing” as a single event of Zucchini-mediated cleavage (see our response to comment #7). We do not intend to measure long trails of Zucchini-dependent piRNA production, which is, as we agree with the reviewer, difficult to measure.

Dowling et al., RNA 2023 reported that a piRNA can slice a target RNA with mismatches at positions g10 and g11, but those instances do not trigger phasing piRNA production. They suggested that only certain slicing events lead to phasing in vivo. On the other hand, our *in-trans* ping-pong analysis measured the perfect matches between g2 and g10. Therefore, the caveat raised by Dowling et al may not affect our claim.

10. The authors should include the grand-childless phenotype observed by Vrettos et al., LSA 2020 in their discussion of maternally inherited piRNAs.

**Response:** Addressed in comment #6
